# Diversity and distribution of thermophilic hydrogenogenic carboxydotrophs revealed by microbial community analysis in sediments from multiple hydrothermal environments in Japan

**DOI:** 10.1007/s00203-019-01661-9

**Published:** 2019-04-27

**Authors:** Kimiho Omae, Yuto Fukuyama, Hisato Yasuda, Kenta Mise, Takashi Yoshida, Yoshihiko Sako

**Affiliations:** 1 Laboratory of Marine Microbiology, Graduate School of Agriculture, Kyoto University, Kitashirakawa Oiwake-cho, Sakyo-ku, Kyoto, 606-8503 Japan; 2 Center for Advanced Marine Core Research, Kochi University, B200 Monobe, Nankoku, Kochi, 783-8502 Japan

**Keywords:** Microbial community analysis, Next-generation sequencing, Hot spring, Thermophile, Carboxydotroph, Hydrogenogen

## Abstract

In hydrothermal environments, carbon monoxide (CO) utilisation by thermophilic hydrogenogenic carboxydotrophs may play an important role in microbial ecology by reducing toxic levels of CO and providing H_2_ for fuelling microbial communities. We evaluated thermophilic hydrogenogenic carboxydotrophs by microbial community analysis. First, we analysed the correlation between carbon monoxide dehydrogenase (CODH)–energy-converting hydrogenase (ECH) gene cluster and taxonomic affiliation by surveying an increasing genomic database. We identified 71 genome-encoded CODH–ECH gene clusters, including 46 whose owners were not reported as hydrogenogenic carboxydotrophs. We identified 13 phylotypes showing > 98.7% identity with these taxa as potential hydrogenogenic carboxydotrophs in hot springs. Of these, Firmicutes phylotypes such as *Parageobacillus*, *Carboxydocella*, *Caldanaerobacter,* and *Carboxydothermus* were found in different environmental conditions and distinct microbial communities. The relative abundance of the potential thermophilic hydrogenogenic carboxydotrophs was low. Most of them did not show any symbiotic networks with other microbes, implying that their metabolic activities might be low.

**Electronic supplementary material:**

The online version of this article (10.1007/s00203-019-01661-9) contains supplementary material, which is available to authorized users.

## Introduction

Hydrothermal systems, where geothermally heated water is expelled through fissures in the Earth’s crust, are located both on land and under the sea. It is now well known that a wide variety of microorganisms, called thermophiles or hyperthermophiles, can prevail and even thrive in such high-temperature environments. The pioneering studies by Brock and his colleagues (Brock 1967; Bott and Brock 1969; Brock and Darland 1970; Brock et al. 1971) at the Yellowstone National Park hot springs established that these organisms grow at near boiling temperatures. Furthermore, a research study led by Pace using molecular phylogenetic techniques demonstrated the high abundance of unidentified thermophilic bacteria and archaea and their remarkable phylogenetic diversity in pink filaments and sediments in the same area (Barns et al. 1994, 1996; Reysenbach et al. 1994; Hugenholtz et al. 1998).

In recent years, microbes that can utilise carbon monoxide (CO) have been found from the hydrothermal area (Sokolova et al. 2009; Techtmann et al. 2009). Although CO is a toxic gas, it can also be a low-potential electron donor and carbon source for many microbes. To date, the list of known thermophilic anaerobic CO-utilizing microorganisms includes acetogenic bacteria (*Moorella thermoacetica*, for instance), sulfate-reducing bacteria (*Desulfotomaculum carboxydivorans*), methanogenic archaea (*Methanothermobacter thermautotrophicus*), and hydrogenogenic bacteria as well as various archaea, such as *Carboxydothermus hydrogenoformans*, *Thermosinus carboxydivorans*, and *Thermococcus* AM4 (Techtmann et al. 2009). Of these, hydrogenogenic bacteria and archaea (collectively designated thermophilic hydrogenogenic carboxydotrophs) are thought to play a key ecological role by virtue of providing a ‘safety valve’ for reducing toxic levels of CO and supplying H_2_ for fuelling H_2_-dependent microbial community processes (Techtmann et al. 2009).

In general, the ability of hydrogenogenic carboxydotrophy is linked to the presence of CO dehydrogenase (CODH)–energy-converting hydrogenase (ECH) gene cluster in genomes. This cluster is believed to be horizontally transferred between the representatives of separate taxa (Techtmann et al. 2012). So far, 28 phylogenetically diverse thermophilic anaerobic hydrogenogenic CO-utilizing archaea and bacteria have been reported (Sokolova et al. 2009; Table 2). Most of them (23 species) are members of the phylum Firmicutes.

In addition to their basic isolation and identification, there are several ecological studies on thermophilic hydrogenogenic carboxydotrophs (Kochetkova et al. 2011; Brady et al. 2015; Yoneda et al. 2015). Notably, a radio isotopic study suggests that the majority of CO is oxidised to CO_2_ (120 μmol L^−1^ of sediment day^−1^) by microbial activities in the hot springs of Uzon Caldera (Kamchatka) (Kochetkova et al. 2011). Thermophilic hydrogenogenic carboxydotrophs of the genera *Carboxydocella* and *Dictyoglomus* have also been isolated from the same environment (Kochetkova et al. 2011). A quantitative polymerase chain reaction (qPCR) analysis targeting the CODH gene, which encodes a key enzyme involved in CO oxidation, suggests that the *Carboxydothermus* species, which is the most studied thermophilic carboxydotrophic species, is widely distributed in a wide range of hydrothermal environments despite its relatively low population size ( ≤ 0.000795% of the total bacterial population) (Yoneda et al. 2015). In addition, using the stable isotope probing (SIP) method by ^13^CO DNA, *Thermincola*, *Desulfotomaculum,* and *Carboxydocella* species were all detected and enriched at geothermal sites, although they are present at < 1% in the original communities (Brady et al. 2015). While there is evidence for the temporal dominance of the *Carboxydothermus* species ( ~ 10% of bacterial population) in hydrothermal environments (Yoneda et al. 2013a), thermophilic hydrogenogenic carboxydotrophs are generally considered to occur in low abundance in the environments.

However, these ecological studies on thermophilic hydrogenogenic carboxydotrophs had a few limitations. Because the sequences of CODH genes are highly diverse, it was difficult to design universal primers that could amplify a wide range of CODH genes from different taxa (Yoneda et al. 2013a). SIP is effective for identifying CO-utilizing microbes in the environment (Brady et al. 2015); however, cultivation bias could be observed. In addition, the previous CO-SIP study was limited to a few neutral pH hot springs (Brady et al. 2015). On the other hand, 16S metagenomics is a culture-independent and high-throughput technique, which is applicable for exploring diverse thermophilic hydrogenogenic carboxydotrophs and co-occurring microbes. The number of available microbial genome sequences has vastly increased thanks to recent advances in next-generation sequencing technology, using which CODH genes were detected in some species that had never been reported to show hydrogenogenic carboxydotrophic growth (Mohr et al. 2018; Inoue et al. 2019a). However, the correlation between the presence of CODH–ECH gene cluster and taxonomic affiliation has not been well understood. Here, we performed a comprehensive survey of a current prokaryotic genomic database and revealed the phylogenetic distribution of CODH–ECH gene clusters across prokaryotes. Next, we performed 16S rRNA gene amplicon (V3/V4 region) sequencing analysis on 100 sediment samples from a wide variety of hydrothermal and mesophilic environments in Japan and unveiled the distribution patterns of these “potential hydrogenogenic carboxydotrophs”.

## Materials and methods

### Sample collection and DNA extraction

We collected a total of 100 sediment samples [17.5 ~ 99.0 °C; pH 2.2 ~ 8.9; oxidation–reduction potential (ORP) − 262 ~  + 449 mV] from terrestrial hydrothermal and mesophilic environments in Japan from May 2014 to March 2017 (Online Resource 1). The sampling sites included 76 on Southern Kyushu Island (Kagoshima prefecture), 14 on Northern Kyushu Island (Oita prefecture), five on the Eastern Izu peninsula (Shizuoka prefecture), and five on the Southern Izu peninsula (Shizuoka prefecture). At the Unagi-onsen hot spring (Southern Kyushu Island), we collected a total of 65 samples in May 2014, May 2015, November 2015, and December 2016 as a previous study suggested that *Carboxydothermus* species are abundant in this environment (Yoneda et al. 2013a). In addition, we previously isolated the *Carboxydocella* strains ULO1 and JDF658 at Unagi-ike lake and the Jiunji-onsen hot spring, respectively (Fukuyama et al. 2017). Temperature was measured using a TX10 digital thermometer (Yokogawa, Tokyo, Japan) with a type K temperature probe (Yokogawa, Tokyo, Japan) at each sampling site. The pH and ORP of the sediment pore water were measured using an HM-31P portable pH meter (DKK-TOA, Tokyo, Japan) with pH (GST-2729C; DKK-TOA, Tokyo, Japan) or ORP (PST-2729C; DKK-TOA, Tokyo, Japan) electrodes. Sediment samples were collected using 50 mL plastic tubes filled with pore water, put into plastic bags with AnaeroPouch-Anaero (Mitsubishi Gas Chemical, Tokyo, Japan), and immediately sealed to minimise contact with oxygen. The samples were then packed in a cooler box with ice, transported to the laboratory, and stored at – 80 °C until use. DNA was extracted from 0.5 g of sediment material using an Extrap Soil DNA Kit Plus ver. 2 (Nippon Steel and SUMIKIN Eco-Tech, Tokyo, Japan) following the manufacturer’s instructions. During the homogenising step, we used a bead beater-type homogeniser, Beads Crusher μT-12 (Taitec, Koshigaya, Japan), at a speed of 3200 r min^−1^ for 60 s. The extracted DNA was stored at – 30 °C until use.

## 16S rRNA gene amplification and sequencing

The V3/V4 region of bacterial and archaeal 16S rRNA genes was amplified with the following prokaryotic universal primer sets (Takahashi et al. 2014): forward (5′-CCTACGGGNBGCASCAG-3′) and reverse (5′-GACTACNVGGGTATCTAATCC-3′) with added overhanging adapter sequences at each 5ʹ-end according to the 16S metagenomic sample preparation guide (https://support.illumina.com/content/dam/illumina-support/documents/documentation/chemistry_documentation/16s/16s-metagenomic-library-prep-guide-15044223-b.pdf). Each sample was amplified with KAPA™ HiFi HotStart ReadyMix (2X) (KAPA Biosystems, South Africa) according to the manufacturer’s instructions. Paired-end (PE, 2 × 300 nucleotides) sequencing was performed with an Illumina MiSeq (MiSeq Reagent kit v2) and followed the manufacturer’s run protocols (Illumina, Inc., San Diego, CA, USA).

## 16S rRNA gene sequence processing and statistical analyses

Primer-binding regions were removed by trimming 17 and 21 nt sequences from the 5′ ends of the forward and reverse reads without adapter regions, respectively, with VSEARCH ver. 2.6.0 (Rognes et al. 2016). The reads were further processed by trimming low-quality regions from the sequences with Trimmomatic ver. 0.36 (SLIDINGWINDOW: 50:20) (Bolger et al. 2014). Using VSEARCH, the paired-end reads were joined and de-multiplexed, and a further round of quality control was conducted to remove sequences shorter than 200 nt as well as those containing ambiguous bases (*N*) or bases with a quality score below 20. Chimeric 16S rDNA sequences were detected using the UCHIME algorithm in the USEARCH package implemented within VSEARCH. The SILVA 132 SSU Ref Nr99 (Quast et al. 2013), a comprehensive, quality checked data sets of small subunit rRNA sequences, was used as a reference for chimera detection. Operational taxonomic units (OTUs) were defined as clusters of sequences that were not singletons (unique sequences that are present exactly once in each sample) with 98.7% similarity using VSEARCH. Then, taxonomic classification of individual OTU was performed with the stand-alone SINA ver. 1.2.11 aligner (Pruesse et al. 2012) using the SILVA 132 SSU Ref Nr99 database as a reference. The non-prokaryotic OTUs (i.e., eukaryote and unclassified domain) were then removed. OTU abundance was estimated by adding prokaryotic singleton reads using the global alignment search option of VSEARCH (–usearch_global—id 0.987), to increase sensitivity. Prior to community analysis, samples with less than 10,000 sequences were omitted (leaving 77 samples) in the beta-diversity patterns. The resulting OTU abundance tables were rarefied to an even number of sequences per sample to ensure equal sampling depth (14,146 sequences per sample) using the vegan package (Oksanen et al. 2017) of the R software (R Core Team 2016). Alpha and beta diversity analyses were then performed with the phyloseq (McMurdie and Holmes 2013) and vegan packages of the R software.

## Database search for CODH–ECH gene clusters

The amino acid sequences corresponding to CODHs were obtained from the Reference Sequence (RefSeq) Database in National Center for Biotechnology Information (NCBI) (December 2018) through a BLASTp search using *C. hydrogenoformans* CooSI (ABB14432.1) subunit as a query. Low-scoring and short-length hits (bit score < 200, amino acid length < 550) including HCPs and partial fragments were excluded from the data set. Then, coding sequences (CDS) within 20 CDSs upstream and downstream of the CODH gene locus were annotated by clusters of orthologous groups of proteins (COGs) (Tatusov 2001) through RPS-BLAST search (*e* value < 10^−6^) using NCBI Conserved Domain Database (Marchler-Bauer et al. 2002). Of these, we identified CODH genes with ECH small and large subunits (COG3260 nd COG3261, respectively) as CODH–ECH gene clusters.

## Phylogenetic analyses

We retrieved the reference 16S rRNA gene sequences that were equal or longer than 1,000 nt and did not include *N* from the genomes of prokaryotes possessing CODH–ECH gene clusters and those that were classified into the same genera as them via the RefSeq genome database. To obtain a non-redundant data set for phylogenetic analysis, retrieved sequences were trimmed into V3/V4 region identical to the amplicons and clustered with 100% similarity using VSEARCH (the sequences utilised in this analysis are listed in Online Resource 6). The sequences were aligned using MAFFT 7.402 (Katoh and Standley 2013). Maximum-likelihood phylogenetic trees were calculated using FastTree ver. 2.1.9 (Price et al. 2010) with an approximate-maximum-likelihood method using the GTR + GAMMA model. Robustness of the topology of the phylogenetic trees was evaluated by local bootstrap values based on 1000 re-samples. The tree was imported into the iTOL online tool (Letunic and Bork 2016) for visualisation.

## Exploring the co-occurrence of thermophilic hydrogenogenic carboxydotrophs and other microbes

Based on the OTU read numbers, a network of phylotype co-occurrence was produced with a minimum Spearman correlation coefficient of 0.8 using R. We retrieved and have presented the smaller networks, including phylotypes, related to the thermophilic hydrogenogenic carboxydotrophs identified in our phylogenetic analysis.

## Results and discussion

### Sample profiles and overview of 16S rRNA gene amplicon sequencing

We collected 100 sediment samples from geographically distant areas in Japan, including Kyushu Island and the Izu Peninsula (Table 1; additional data are provided in Online Resource 1). Except for a single sample from Unagi-ike lake, which has a moderate environment (17.5 °C; pH 7.37; ORP, + 75 mV), all the samples were collected from geothermally heated hydrothermal environments (33.8–99.0 °C). Although the in situ environmental conditions of the sampling sites were variable, the hot springs on Kyushu Island had an acidic pH [average pH 4.1 ± 1.1 (sd); measurable sites, *n* = 82], whereas those on the Izu Peninsula were neutral or weakly alkaline (pH 8.3 ± 0.4; *n* = 10).

**Table 1:** Summary of samples

Sampling area and time point	Sampling date	Numbers of samples	Temperature (°C)	pH	ORP (mV)	Salinity (%)
1405_Unagi	May 2014	13	41.4 ~ 99.0	2.2 ~ 4.9	− 218 ~ + 426	n.m
1505_Unagi	May 2015	15	33.8 ~ 95.8	4.4 ~ 5.8	− 174 ~ + 277	n.m
1511_Unagi	November 2015	19	41.2 ~ 96.2	3.3 ~ 5.6	− 130 ~ + 449	n.m
1612_Unagi	December 2016	18	35.5 ~ 96.9	2.6 ~ 5.9	− 262 ~ + 164	n.m
1612_Kirishima	December 2016	10	63.4 ~ 88.7	2.4 ~ 4.1	− 179 ~ + 310	0
1703_Komatsu	March 2017	14	61.1 ~ 80.9	2.2 ~ 5.6	− 179 ~ + 286	n.m
1501_Eastern_Izu	January 2015	5	68.2 ~ 80.1	8.4 ~ 8.5	− 22 ~ + 189	0.0 ~ 0.2
1501_Southern_Izu	January 2015	5	60.1 ~ 78.5	7.7 ~ 8.9	− 30 ~ + 259	0.0 ~ 2.4
1612_Unagi-ike_lake	December 2016	1	17.5	7.37	75	n.m

*n.m.* not measured

Our 16S rRNA gene amplicon sequencing analysis generated 8,531,132 bacterial and archaeal quality-controlled sequences from the 100 samples, with a range of 107–398,919 sequences (average, 85,311 sequences) per sample (Online Resource 1). A total of 9,394 prokaryotic OTUs were defined at the 98.7% similarity level, and 23–4,737 OTUs (average, 299 OTUs) were observed in each sample (Online Resource 2). Diversity analysis using rarefied 77 samples with equal or greater than 10,000 sequences revealed that microbial communities in the sampled hot springs showed much lower alpha diversity than those in the moderate environment (Unagi-ike lake; Online Resource 3), indicating that high temperature imposed constraints on community properties as observed in other studies (Sharp et al. 2014).

**Fig. 1:**
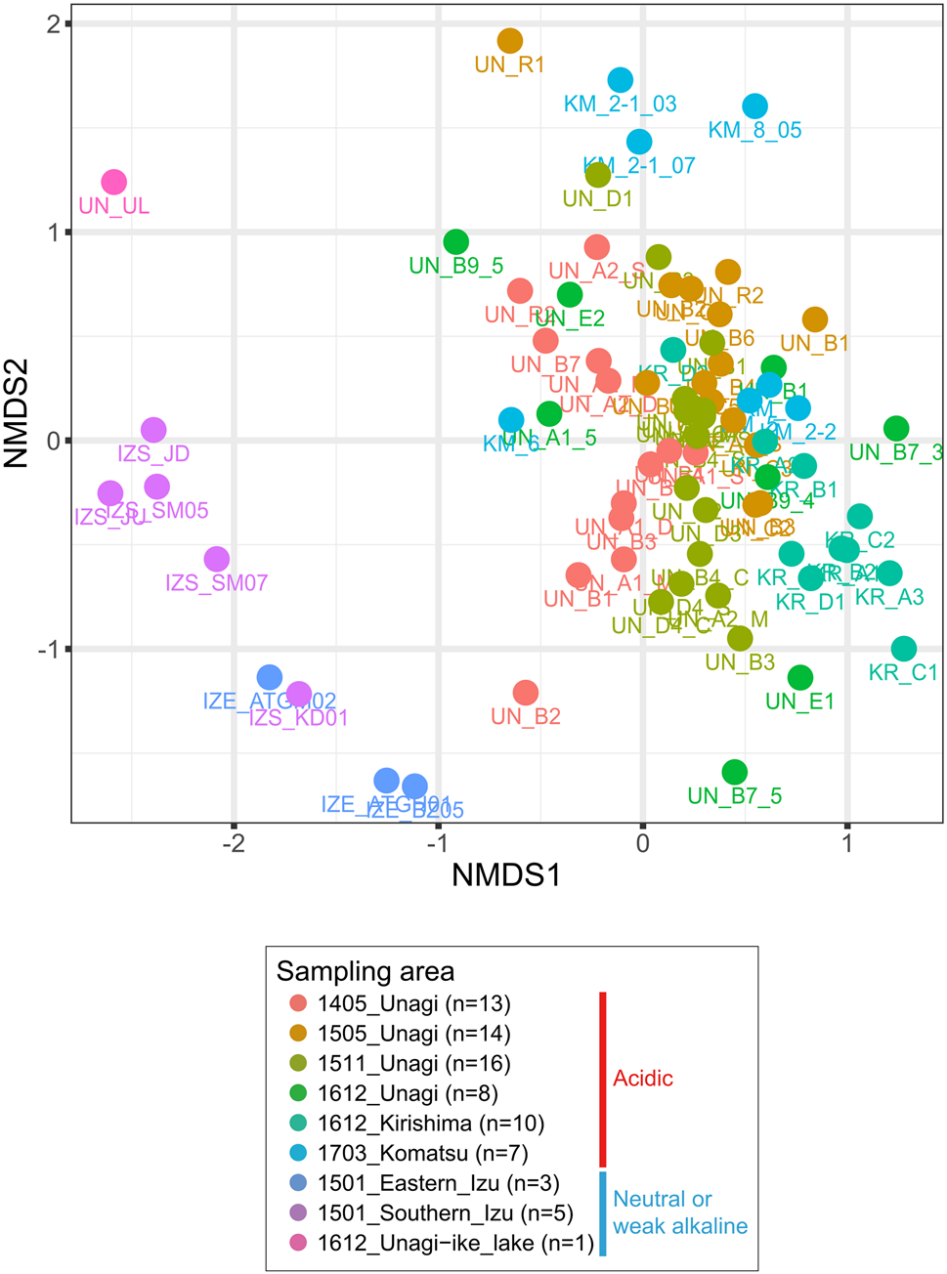
Non-metric multidimensional scaling analysis based on Bray–Curtis dissimilarity. Plot colours illustrate sampling area and period

Furthermore, our beta diversity analysis revealed apparent differences between the acidic hot springs on Kyushu Island and the neutral or weak alkaline environments on Izu Peninsula and Unagi-ike lake (Fig. 1). At the domain level, microbial communities in the acidic hot springs were dominated by archaea, whereas those in the neutral or weak alkaline environments were dominated by bacteria (Online Resource 4). The phylotypes that shared 100% identity with *Vulcanisaeta souniana* (phylum Crenarchaeota; OTU_1) and *Thermus thermophilus* (phylum Deinococcus-Thermus; OTU_20) were notably prominent in the acidic hot springs and neutral or weak alkaline environments, respectively. *V. souniana* is a heterotrophic anaerobic hyperthermophilic crenarchaeote found in hot springs that grows optimally at 85–90 °C and pH 4.0–4.5 (Itoh et al. 2002). In contrast, *T.thermophilus* is an extremely thermophilic bacterium also found in hot springs, but its optimal growth occurs at 65–72 °C and pH 7.5 (Oshima and Imahori 1974). Although the major phylotypes were the same in each acidic and neutral or weak alkaline environments, our non-metric multidimensional scaling analysis using rarefied 77 samples with greater equal than 10,000 sequences shows that microbial community compositions vary across each sampling sites (Fig. 1).

## CODH–ECH gene clusters found in prokaryotic genomes

A previous study examined CODHs and their genomic context in 2887 microbial genomes and revealed 185 genomes that encoded at least one CODH gene (Techtmann et al. 2012). Of these, 12 genomes possessed CODH–ECH gene clusters. However, by December 2018, the number of sequenced microbial genome entries in the RefSeq genome database had reached 142,909, and novel thermophilic hydrogenogenic carboxydotrophs had been reported. Therefore, we searched CODH–ECH gene clusters in the current RefSeq database and examined their taxonomic information. We identified 71 genomes encoding CODH–ECH gene clusters, which include 40 thermophile genomes (14 genera), 25 mesophile genomes (12 genera), and six unclassified microbial genomes (Table 2; additional data are listed in Online Resource 5). All mesophilic members were classified into the phylum Proteobacteria, which included phototrophic bacteria or sulfate-reducing bacteria, whereas thermophilic members were phylogenetically diverse and classified into the phylum Crenarchaeota, Euryarchaeota, and Firmicutes. Of these 71 genomes, 46 have never been reported as hydrogenogenic carboxydotrophs (Table 2), and the presence of CODH–ECH gene clusters in 22 genomes was reported for the first time in this study (Online Resource 5).

**Table 2:** Prokaryotes possessing CODH–ECH gene clusters

Organism	Hydrogenogenic carboxydotrophic growth	Isolation source	
		Type	References
**Crenarchaeota (thermophilic)**			
*Thermofilum carboxyditrophus* 1505	Yes (Sokolova et al. 2009)	Water and mud	Sokolova et al. (2009)
**Euryarchaeota (thermophilic)**			
*Thermococcus barophilus* CH5	Yes (Kozhevnikova et al. 2016)	Deep-sea hydrothermal fields	Kozhevnikova et al. (2016)
*Thermococcus barophilus* MP	Yes (Kozhevnikova et al. 2016)	Deep-sea hydrothermal fields	Kozhevnikova et al. (2016)
*Thermococcus guaymasensis* DSM 11113	n.r	Hydrothermal vent sediment	Canganella et al. (1998)
*Thermococcus onnurineus* NA1	Yes (Bae et al. 2006)	Deep-sea hydrothermal fields	Bae et al. (2006)
*Thermococcus paralvinellae* ES1	n.r	Active hydrothermal vent chimneys	Hensley et al. (2014)
*Thermococcus* sp. AM4	Yes (Sokolova et al. 2004b)	Active chimney	Sokolova et al. (2004b)
**Firmicutes (thermophilic)**			
*Parageobacillus thermoglucosidasius* B4168	n.r	n.r	n.r
*Parageobacillus thermoglucosidasius* C56-YS93	n.r	n.r	n.r
*Parageobacillus thermoglucosidasius* DSM 2542^a^	Yes (Mohr et al. 2018)	n.r	Suzuki et al. (1983)
*Parageobacillus thermoglucosidasius* GT23	n.r	n.r	n.r
*Parageobacillus thermoglucosidasius* NBRC 107763	n.r	n.r	n.r
*Parageobacillus thermoglucosidasius* NCIMB 11955	n.r	n.r	n.r
*Parageobacillus thermoglucosidasius* TG4	Yes (Inoue et al. 2019b)	Marine sediment	Inoue et al. (2019a)
*Parageobacillus thermoglucosidasius* TM242	n.r	n.r	n.r
*Parageobacillus thermoglucosidasius* TNO-09.020	n.r	n.r	n.r
*Parageobacillus thermoglucosidasius* Y4.1MC1	n.r	n.r	n.r
*Carboxydocella* sp. JDF658	Yes (Fukuyama et al. 2017)	Open-air stream from a hot spring well	Fukuyama et al. (2017)
*Carboxydocella* sp. ULO1	Yes (Fukuyama et al. 2017)	Sediment of a maar lake	Fukuyama et al. (2017)
*Carboxydocella sporoproducens* DSM 16521	Yes (Slepova et al. 2006)	Hot spring	Slepova et al. (2006)
*Carboxydocella thermautotrophica* 019	Yes (Toshchakov et al. 2018)	Thermal field	Toshchakov et al. (2018)
*Carboxydocella thermautotrophica* 041	Yes (Sokolova et al. 2002)	Terrestrial hot vent	Sokolova et al. (2002)
*Desulfosporosinus* sp. OL	n.r	n.r	n.r
*Desulfotomaculum nigrificans* CO-1-SRB	Yes (Parshina et al. 2005)	Anaerobic bioreactor sludge	Sokolova et al. (2009)
*Thermincola ferriacetica* Z-0001	Yes (Zavarzina et al. 2007)	Ferric deposits of a terrestrial hydrothermal spring	Zavarzina et al. (2007)
*Thermincola potens* JR	Yes (Wrighton et al. 2008; Byrne-Bailey et al. 2010)	Thermophilic microbial fuel cell	Wrighton et al. (2008); Byrne-Bailey et al. (2010)
*Caldanaerobacter subterraneus* subsp. *pacificus* DSM 12653	Yes (Sokolova et al. 2001; Fardeau et al. 2004)	Oilfields	Fardeau et al. (2004)
*Caldanaerobacter subterraneus* subsp. *tengcongensis* MB4	n.r	Oilfields	Fardeau et al. (2004)
*Caldanaerobacter subterraneus* subsp. *yonseiensis* KB-1	n.r	Oilfields	Fardeau et al. (2004)
*Calderihabitans maritimus* KKC1	Yes (Yoneda et al. 2013b)	Submerged marine caldera	Yoneda et al. (2013b)
*Carboxydothermus hydrogenoformans* Z-2901	Yes (Svetlichny et al. 1991)	Hot swamp	Svetlichny et al. (1991)
*Carboxydothermus islandicus* SET	Yes (Novikov et al. 2011)	Hot spring	Novikov et al. (2011)
*Moorella glycerini* NMP	n.r	Underground gas storage	Slobodkin et al. (1997)
*Moorella* sp. Hama-1	n.r	Thermophilic anaerobic digestion reactor	Harada et al. (2018)
*Moorella stamsii* DSM 26271	Yes (Alves et al. 2013)	Anaerobic sludge	Alves et al. (2013)
*Moorella thermoacetica* DSM 21394	Yes (Jiang et al. 2009)	Anaerobic bioreactors	Jiang et al. (2009)
*Thermanaeromonas toyohensis* ToBE	n.r	Geothermal aquifer in mine	Mori et al. (2002)
*Thermoanaerobacter* sp. YS13	n.r	Geothermal hot spring	Peng et al. (2016)
*Thermosinus carboxydivorans* Nor1	Yes (Sokolova et al. 2004a)	Hot spring	Sokolova et al. (2004a)
**Proteobacteria (mesophilic)**			
*Rhodopseudomonas palustris* BisB18	n.r	River sediment	Oda et al. (2008)
*Pleomorphomonas carboxyditropha* SVCO-16	n.r	Anaerobic sludge	Esquivel-Elizondo et al. (2018)
*Pseudovibrio* sp. POLY-S9	n.r	Intertidal marine sponge	Alex and Antunes (2015)
*Pseudovibrio* sp. Tun.PSC04-5.I4	n.r	n.r	n.r
*Rhodospirillum rubrum* ATCC 11170	Yes (Kerby et al. 1992)	Fresh water	Munk et al. (2011)
*Rhodospirillum rubrum* F11	Yes (Singer et al. 2006)	n.r	n.r
*Desulfovibrio bizertensis* DSM 18034	n.r	Marine sediment	Haouari et al. (2006)
*Pseudodesulfovibrio piezophilus* C1TLV30	n.r	Wood falls at deep sea	Khelaifia et al. (2011)
*Geobacter bemidjiensis* Bem	n.r	Subsurface sediments	Nevin et al. (2005)
*Geobacter pickeringii* G13	n.r	Kaolin clays	Shelobolina et al. (2007)
*Ferrimonas futtsuensis* DSM 18154	n.r	Sediment	Nakagawa et al. (2006)
*Ferrimonas kyonanensis* DSM 18153	n.r	Alimentary tract of littleneck clams	Nakagawa et al. (2006)
*Ferrimonas sediminum* DSM 23317	n.r	Coastal sediment	Ji et al. (2013)
*Shewanella* sp. M2	n.r	n.r	n.r
*Shewanella* sp. R106	n.r	n.r	n.r
*Citrobacter amalonaticus* Y19	Yes (Oh et al. 2008)	Anaerobic wastewater sludge digester	Jung et al. (1999)
*Salmonella enterica* subsp. *enterica* serovar Montevideo 50262	n.r	n.r	n.r
*Salmonella enterica* subsp. *enterica* serovar Montevideo 50270	n.r	n.r	n.r
*Salmonella enterica* subsp. *enterica* serovar Senftenberg 50263	n.r	n.r	n.r
*Salmonella enterica* subsp. *enterica* serovar Senftenberg 50264	n.r	n.r	n.r
*Salmonella enterica* subsp. *enterica* serovar Senftenberg 50265	n.r	n.r	n.r
*Salmonella enterica* subsp. *enterica* serovar Senftenberg 50271	n.r	n.r	n.r
*Salmonella enterica* subsp. *enterica* serovar Senftenberg 50272	n.r	n.r	n.r
*Salmonella enterica* subsp. *enterica* serovar Senftenberg SS209	n.r	n.r	n.r
*Photobacterium marinum* AK15	n.r	Sediment	Srinivas et al. (2013)
**Uncultured**		n.r	n.r
Candidatus Korarchaeota archaeon MDKW	n.r	Hot springs metagenomes	n.r
Clostridiales bacterium DRI-13	n.r	Subglacial ecosystem	n.r
Rhizobiales bacterium AFS016371	n.r	Soil	n.r
Rhizobiales bacterium AFS041951	n.r	Soil	n.r
Rhizobiales bacterium AFS049984	n.r	Soil	n.r
Rhizobiales bacterium AFS089140	n.r	Soil	n.r

*n.r.* not reported

^a^Two genomes are available for this strain in the database

**Fig. 2:**
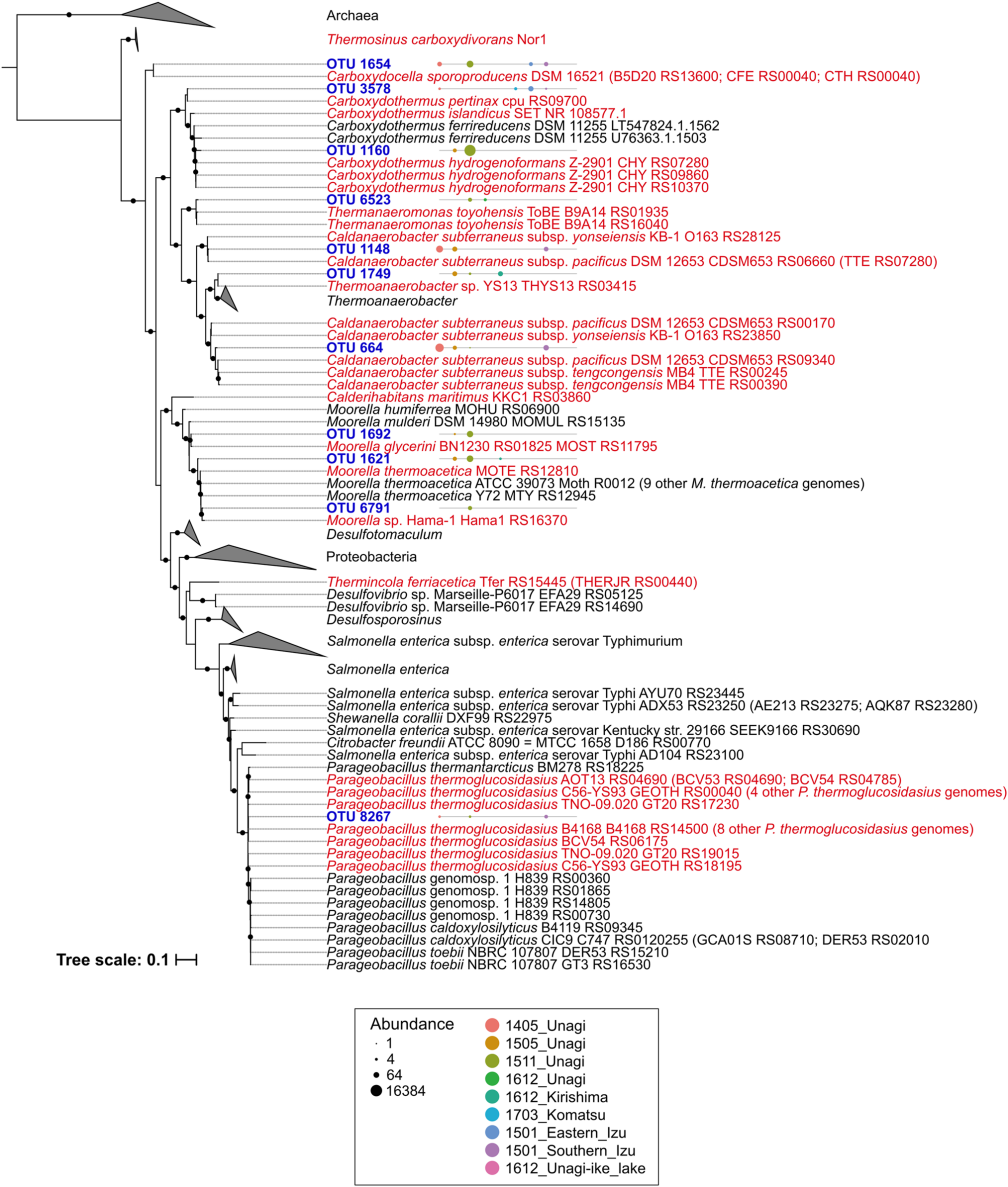
Phylogenetic reconstruction of potential thermophilic hydrogenogenic carboxydotrophic phylotypes of the Phylum Firmicutes. The 16S rRNA sequences used in this analysis are listed in Online Resource 6. Other but identical sequences to their leaves are shown in parenthesis (only one sequence per genome are shown). The phylotype sequences obtained in this study are expressed by ‘operational taxonomic unit (OTU)’ prefix. Microbes possessing CODH − ECH gene clusters and *Carboxydothermus pertinax* (cpu_RS09700) are shown in red font. Nodes supported by a bootstrap value greater than 80% are indicated by black circles. The bubble plots which are shown at the right of OTUs display the distribution pattern of each phylotype. Abundance is indicated by the number of amplicon reads in each sample

Conservation patterns of CODH–ECH gene clusters were different in each genus (Fig. 2). We classified these genera into three groups: (1) the CODH–ECH gene clusters and the hydrogenogenic carboxydotrophy ability were well conserved; (2) a portion of members conserved the CODH–ECH gene clusters; and (3) genera that we could not classify into (1) nor (2) because of inadequate availability of genomic information. *Thermincola*, *Carboxydocella*, *Carboxydothermus*, and *Caldanaerobacter* were classified into the group (1). In most cases, the phylogeny of CODH genes was corresponding to their taxonomic phylogeny in this group (Adam et al. 2018; Fukuyama et al. 2018; Toshchakov et al. 2018), suggesting that the CODH–ECH gene clusters descended from the common ancestors of each genus. The genus *Carboxydothermus* has been one of the most studied models of thermophilic carboxydotrophy, and the members of this genus possess four or five CODH genes (Fukuyama et al. 2018). A comparative genomic analysis in *Carboxydothermus* revealed that the CODH–ECH gene clusters were conserved in the members except for *C. pertinax*, which lacked only the CODH (CODH-I) unit of CODH–ECH gene cluster and *Carboxydothermus ferrireducens*, which lacked the whole CODH–ECH gene cluster (Fukuyama et al. 2018). *C. ferrireducens* can grow carboxydotrophically, but is not hydrogenogenic (Slobodkin et al. 2006). On the other hand, *C.pertinax* can grow by hydrogenogenic carboxydotrophy (Yoneda et al. 2012), and it is suggested that *C. pertinax* could couple alternative CODH (CODH-II) to the distal ECH (Fukuyama et al. 2018). *C. pertinax* was the only isolate that could grow by hydrogenogenic carboxydotrophy without the CODH–ECH gene cluster. *Caldanaerobacter subterraneus* subspecies can oxidise CO and possess CODH–ECH gene clusters, whose structures are very similar (Sant’Anna et al. 2015). However, phylogenetic reconstruction of CODH genes revealed that CODH genes from *C. subterraneus* have distinct evolutionary histories. It is suggested that replacement of CODH gene occurred by a horizontal gene transfer event in *C. subterraneus* subsp. *tengcongensis* and *C. subterraneus* subsp. *yonseiensis* (Sant’Anna et al. 2015). *Thermococcus*, *Thermofilum*, *Thermoanaerobacter*, *Moorella*, *Desulfotomaculum*, *Desulfosporosinus*, *Parageobacillus*, and members of the phylum Proteobacteria were classified into group (2). Because most species of *Thermococcus*, *Thermofilum*, *Thermoanaerobacter*, *Desulfotomaculum*, and *Desulfosporosinus* did not possess the CODH–ECH gene clusters, it was suggested that CODH–ECH gene clusters might have been obtained by a portion of the members in a horizontal gene transfer event. In fact, this cluster is believed to be horizontally transferred between the representatives of separate taxa (Techtmann et al. 2012). In the genus *Moorella*, *Moorella stamsii* and *Moorella glycerini* possessed identical CODHs that were flanked by ECH gene clusters. *Moorella* sp. Hama-1 and *Moorella thermoacetica* DSM 21394, which formed a different subclade from *M. stamsii* and *M. glycerini*, also possessed a similar CODH–ECH gene cluster. However, it was revealed that the other 11 *M. thermoacetica* strains did not possess the CODH–ECH gene cluster (Online Resource 5). *M. thermoacetica* might be an acetogenic carboxydotroph rather than being hydrogenogenic, as reported previously (Pierce et al. 2008; Schuchmann and Müller 2014), and only strain DSM 21394 might be hydrogenogenic. *Parageobacillus thermoglucosidasius* is the only facultative anaerobic bacillus among the thermophilic hydrogenogenic carboxydotrophic species (Mohr et al. 2018). Although other *Parageobacillus* species did not possess the CODH–ECH gene cluster, *P. thermoglucosidasius* possesses a CODH–ECH gene cluster that is phylogenetically related to those of *Moorella* and *Caldanaerobacter* (Mohr et al. 2018). Unlike *M. thermoacetica*, all 10 genomes of *P. thermoglucosidasius* have conserved the CODH–ECH gene clusters (Online Resource 5), and hydrogenogenic carboxydotrophy might be an important trait for this species. The other species, *Thermanaeromonas toyohensis*, *Thermosinus carboxydivorans*, *Calderihabitans maritimus*, and uncultured archaea and bacteria (Candidatus Korarchaeota archaeon MDKW, Clostridium bacterium DRI-13, and Rhizobiales bacterium) were classified into the group (3).

## Diversity and distribution of thermophilic hydrogenogenic carboxydotrophs

In the 16S amplicon sequencing analysis, we revealed that the representative sequences of 13 phylotypes showed > 98.7% identity with known thermophilic hydrogenogenic carboxydotrophs or microbes possessing CODH–ECH gene clusters, and 10 phylotypes were members of the phylum Firmicutes (Fig. 2, Online Resource 7). Of these, the representative sequences of OTU_1654 and OTU_3578 were identical to *Carboxydocella* species and *C. pertinax*, respectively, and OTU_664 and OTU_1148 showed 98.8% and 99.5% identities with *C. subterraneus* subspecies, respectively. They were members of group (1). It should be noted that the abundant phylotype OTU_1160 showed 97.7% identity with *Carboxydothermus* species, all of which possess multi CODH gene clusters. The phylotypes that were close to *Thermofilum carboxyditrophus* 1505 (OTU_1051, identity = 99%), *M. thermoacetica* DSM 21394 (OTU_1621, identity = 98.8%; OTU_6791, identity = 99.1%), *M. glycerini* DSM 26271 or *M. stamsii* NMP (OTU_1692, identity = 99.3%), *Thermoanaerobacter* sp. YS13 (OTU_1749, identity = 100%), *Thermococcus barophilus* (OTU_1816, identity = 99%), *T. toyohensis* ToBE (OTU_6523, identity = 99.3%), and *P. thermoglucosidasius* (OTU_8267, identity = 100%), were members of group (2) hydrogenogenic carboxydotrophs, suggesting that these phylotypes are also potential thermophilic hydrogenogenic carboxydotrophs. We also found that OTU_1000 showed 99% identity with Candidatus Korarchaeota archaeon MDKW, whose genome was assembled from Washburn Hot Spring metagenome.

The 13 phylotypes of potential thermophilic hydrogenogenic carboxydotrophs were detected in 45 samples (Fig. 2, Online Resource 7). Of these, OTU_1654 (*Carboxydocella*), OTU_664 (*C. subterraneus*), OTU_1148 (*C. subterraneus*), OTU_3578 (*C. pertinax*), and OTU_8267 (*P. thermoglucosidasius*) were detected in 7 to 21 samples and widely distributed in geographically distinct areas (both Kyushu Island and the Izu Peninsula) that showed different environmental conditions and microbial community structures (Fig. 2, Online Resource 7). OTU_1000, uncultured archaeon phylotype, was also detected widely from 11 samples. The distribution of OTU_1051 (*T. carboxyditrophus*), OTU_1692 (*M. glycerini* or *M. stamsii*), OTU_1749 (*Thermoanaerobacter* sp. YS13), OTU_6523 (*T. toyohensis*), OTU_1621 (*M. thermoacetica* DSM 21394), and OTU_6791 (*M. thermoacetica* DSM 21394) was limited to hot springs in Kyushu Island (mainly in Unagi-onsen in May 2015, November 2015, and December 2016). OTU_1816, the phylotype of *T. barophilus* that was isolated from a deep-sea hydrothermal vent (Marteinsson et al. 1999), was uniquely detected in the saline hot springs in the Izu Peninsula (Online Resource 7).

In most cases, the phylotypes of potential thermophilic hydrogenogenic carboxydotrophs showed a relative abundance of < 0.1%. Previous studies also suggested that Firmicutes carboxydotroph abundance in hydrothermal environments is usually low (Brady et al. 2015; Yoneda et al. 2015). However, the phylotypes of *C. subterraneus* (OTU_664), *Carboxydocella* (OTU_1654), *C. pertinax* (OTU_3578), and *Carboxydothermus* phylotype (OTU_1160) exhibited a relative abundance of > 0.1% in nine samples (Online Resource 7). In particular, we found that the relative abundance of OTU_1654 reached 8.47% per sample at the 1511_UN_A2_D site (70.9 °C, pH 4.68). OTU_1160 was abundant in Unagi-onsen in November 2015, and its relative abundance reached 7.75% and 11% at the 1511_UN_A2_D and 1511_UN_B4_C (94.9 °C, pH 3.65) sites, respectively. However, we could not identify whether the phylotypes, whose relative abundance exceeded 0.1% were growing in these environments, because six of the nine sites showed higher temperature or lower pH than the growth conditions for the isolates of *C. subterraneus* subspecies (50–80 °C, pH 4.5–9.0) (Fardeau et al. 2004), *Carboxydocella* species (40–70 °C, pH 6.2–8.0) (Sokolova et al. 2002; Slepova et al. 2006; Toshchakov et al. 2018), and *Carboxydothermus* species (40–78 °C, pH 4.6 − 8.6) (Svetlichny et al. 1991; Novikov et al. 2011; Yoneda et al. 2012) (Online Resource 8). The other three sites including 1511_UN_A2_D showed moderate environmental conditions, where the growth could occur (Online Resource 8), but the DNA yields from these sites were low ( < 15 ng/g sediment). Firmicutes members of *Carboxydothermus*, *Carboxydocella,* and *Caldanaerobacter* are reported to be able to form endospore (Kim et al. 2001; Wu et al. 2005; Slepova et al. 2006). Notably, these groups possessed the genes for endospore formation. It was speculated that DNAs of these phylotypes might persist in such environments longer than those of non-spore-forming prokaryotes.

Carboxydotrophs have been suggested to be functionally important, because they mediate a ‘currency exchange’ between CO and hydrogen in hydrothermal environments (Techtmann et al. 2009). For example, symbiotic interactions have been observed between *C. hydrogenoformans* and thermophilic sulfate reducers in culture, wherein the carboxydotroph provides protection from CO toxicity, whereas H_2_ is removed by sulfate reduction, thus reducing end-product inhibition (Parshina et al. 2005). We investigated the co-occurrence of the potential thermophilic hydrogenogenic carboxydotrophs and other microbes using non-parametric Spearman correlations of phylotype presence/absence across all sampling sites. Among the phylotypes present in at least seven sites, networks between OTU_664 and four uncultured microbes, and between OTU_1000 and two uncultured bacteria were identified with a Spearman correlation coefficient > 0.8 (Online Resource 9). There seem to be no specific symbiotic interactions between most of the potentially hydrogenogenic carboxydotrophic phylotypes and other microbes at these sampling sites.

A microbial population whose relative abundance is < 0.1% is called ‘rare biosphere’ and contributes to a persistent microbial seed bank, which is a collection of dormant microorganisms that can respond to favourable environmental conditions (Lynch and Neufeld 2015). Endospore formation has an important role for dormancy as well as microbial dispersal (Hubert et al. 2009; Müller et al. 2013; Zeigler 2014; Lynch and Neufeld 2015). It was considered that Firmicutes members of the potential thermophilic hydrogenogenic carboxydotrophs found in a variety of hot springs (in most case, as rare biosphere) might form endospores in extreme environmental conditions and have a strategy of microbial seed bank dynamics. The result that most of the potential hydrogenogenic carboxydotrophs did not show any symbiotic networks with other microbes also might support the speculation that metabolic activities of these members are low in extreme environments.

## Conclusion

This study explored the distribution, diversity, and ecology of thermophilic carboxydotrophs across various hydrothermal environments using microbial community analysis. First, we searched CODH–ECH gene clusters in the current microbial genomic database and revealed 71 genomes encoding CODH–ECH gene clusters. Of these, 46 were genomes whose carriers have never been reported as hydrogenogenic carboxydotrophs. In a microbial community analysis, we identified 13 phylotypes that showed > 98.7% identity with thermophilic members of these taxa. Of these, 10 phylotypes were members of the phylum Firmicutes, and *Parageobacillus*, *Carboxydocella*, *Caldanaerobacter*, and *Carboxydothermus* phylotypes were found across geographically distant hot springs with different environmental conditions, wherein distinct microbial community structures were formed. Although the relative abundance of the *Carboxydothermus* and *Carboxydocella* phylotypes was greater than 1% at some sites, most of the potentially thermophilic hydrogenogenic carboxydotrophs were usually rare biospheres, whose relative abundances were < 0.1%. They might be in dormant states in extreme environmental conditions. Although symbiotic interactions between hydrogenotrophic microbes and hydrogenogenic carboxydotrophs have been suggested (Parshina et al. 2005), no symbiotic interaction was identified between most of these phylotypes and other microbes in our study, leading to the speculation that thermophilic hydrogenogenic carboxydotrophic species might not be active in these environments. However, the previous sediment incubation and cultivation studies have shown that *Carboxydothermus* and *Carboxydocella* species respond to the presence of CO and actively grow (Kochetkova et al. 2011; Yoneda et al. 2012 , 2015; Brady et al. 2015). There is also evidence that an unusually high-density population (equivalent to 9.45 × 10^5^ cells g sediment^−1^) of *Carboxydothermus* is present in Unagi-onsen hot springs (Yoneda et al. 2013a), suggesting that they are viable in the environment. While further studies such as transcription analysis are needed to better understand the ecological function of thermophilic hydrogenogenic carboxydotrophs, the present study provides essential information concerning their distribution and diversity in a variety of volcanic environments.

## Electronic supplementary material

Below is the link to the electronic supplementary material.

Supplementary file2 (XLSX 30 kb)

Supplementary file3 (XLSX 5664 kb)

Supplementary file4 (PDF 317 kb)

Supplementary file5 (PDF 521 kb)

Supplementary file6 (XLSX 21 kb)

Supplementary file7 (XLSX 735 kb)

Supplementary file8 (XLSX 22 kb)

Supplementary file9 (PDF 61 kb)

Supplementary file1 (XLSX 17 kb)
