# Supplementary material for: Diversity and distribution of thermophilic hydrogenogenic carboxydotrophs revealed by microbial community analysis in sediments from multiple hydrothermal environments in Japan
Source: Arch Microbiol. 2019 Apr 27;201(7):969–82. doi: 10.1007/s00203-019-01661-9 (PMC6687684; doi:10.1007/s00203-019-01661-9)
Supplement: Supplementary file 3 — Supplementary file4 (PDF 317 kb) [file 203_2019_1661_MOESM3_ESM.pdf]

"Diversity and distribution of thermophilic hydrogenogenic carboxydotrophs revealed by microbial community analysis in sediments from multiple hydrothermal environments in Japan"

Arch. Microbiol. Kimiho Omae, Yuto Fukuyama, Hisato Yasuda, Kenta Mise, Takashi Yoshida, and Yoshihiko Sako*
Laboratory of Marine Microbiology, Graduate School of Agriculture, Kyoto University; sako@kais.kyoto-u.ac.jp

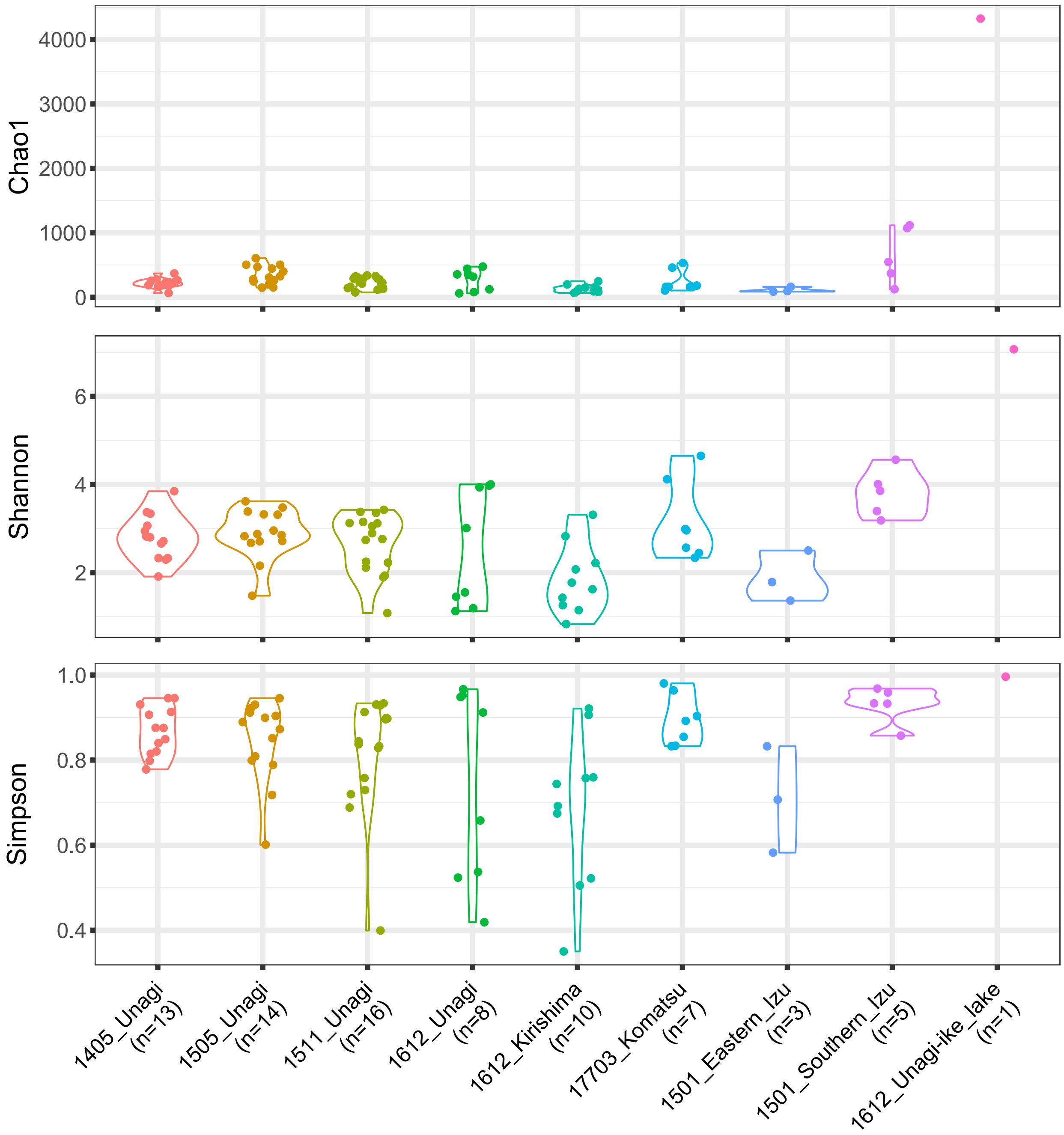

Online Resource 3 Alpha diversity using rarefied samples. The upper, middle, and bottom panels display the Chao1, Shannon, and Simpson indices, respectively. Samples from different areas and periods were plotted separately.
