# Supplementary material for: Diversity and distribution of thermophilic hydrogenogenic carboxydotrophs revealed by microbial community analysis in sediments from multiple hydrothermal environments in Japan
Source: Arch Microbiol. 2019 Apr 27;201(7):969–82. doi: 10.1007/s00203-019-01661-9 (PMC6687684; doi:10.1007/s00203-019-01661-9)
Supplement: Supplementary file 4 — Supplementary file5 (PDF 521 kb) [file 203_2019_1661_MOESM4_ESM.pdf]

"Diversity and distribution of thermophilic hydrogenogenic carboxydotrophs revealed by microbial community analysis in sediments from multiple hydrothermal environments in Japan"

Arch. Microbiol. Kimiho Omae, Yuto Fukuyama, Hisato Yasuda, Kenta Mise, Takashi Yoshida, and Yoshihiko Sako*
Laboratory of Marine Microbiology, Graduate School of Agriculture, Kyoto University; sako@kais.kyoto-u.ac.jp

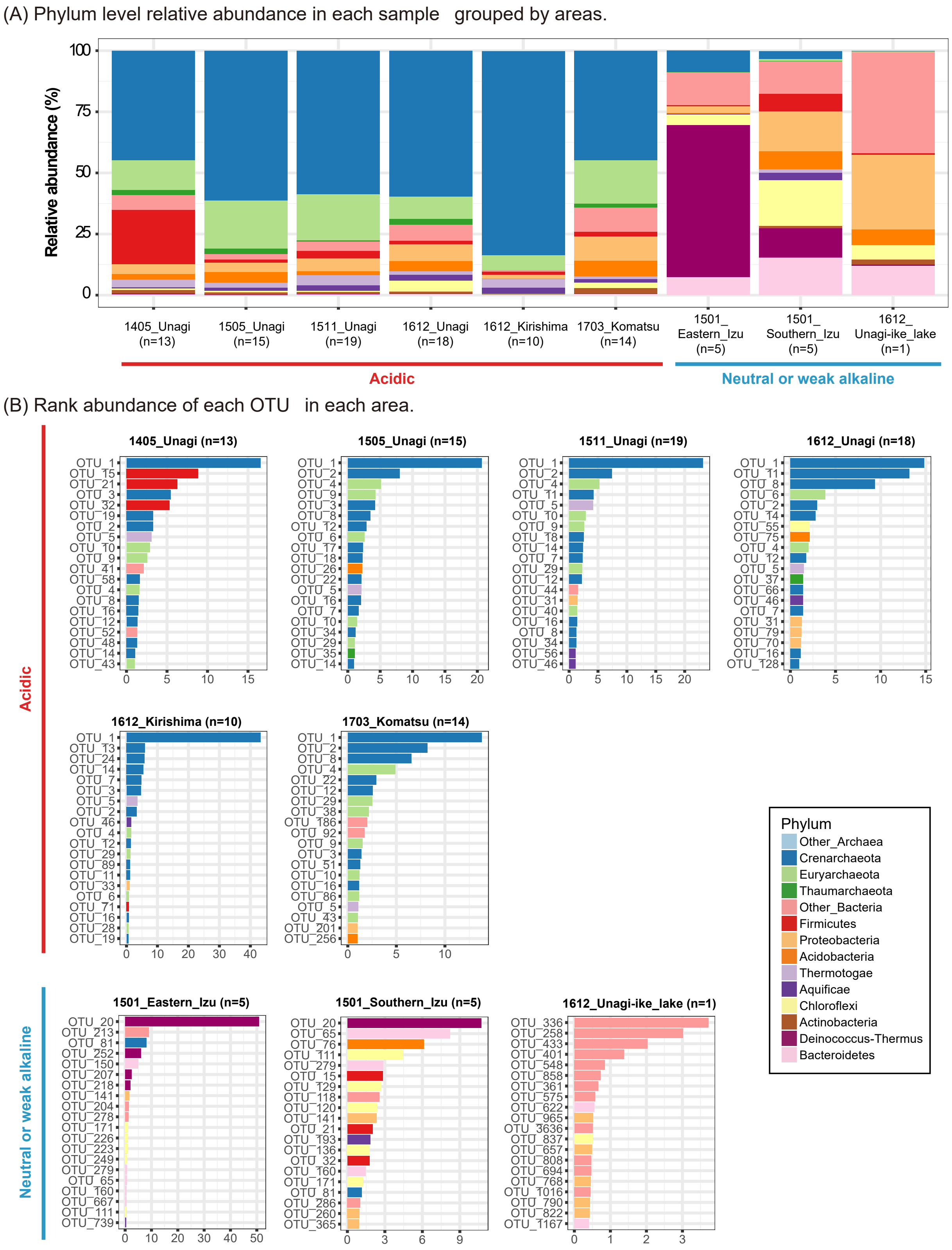

Online Resource 4 Microbial taxonomic compositions in each area and period. (A) Phylum level community composition of each area. (B) The proportion of the 20 most predominant operational taxonomic units (OTUs) in each community. Bar colours indicate the phyla to which individual groups were assigned.
