# Supplementary material for: Diversity and distribution of thermophilic hydrogenogenic carboxydotrophs revealed by microbial community analysis in sediments from multiple hydrothermal environments in Japan
Source: Arch Microbiol. 2019 Apr 27;201(7):969–82. doi: 10.1007/s00203-019-01661-9 (PMC6687684; doi:10.1007/s00203-019-01661-9)
Supplement: Supplementary file 8 — Supplementary file9 (PDF 61 kb) [file 203_2019_1661_MOESM8_ESM.pdf]

"Diversity and distribution of thermophilic hydrogenogenic carboxydotrophs revealed by microbial community analysis in sediments from multiple hydrothermal environments in Japan"

*Arch. Microbiol.* Kimiho Omae, Yuto Fukuyama, Hisato Yasuda, Kenta Mise, Takashi Yoshida, and Yoshihiko Sako*
Laboratory of Marine Microbiology, Graduate School of Agriculture, Kyoto University; sako@kais.kyoto-u.ac.jp

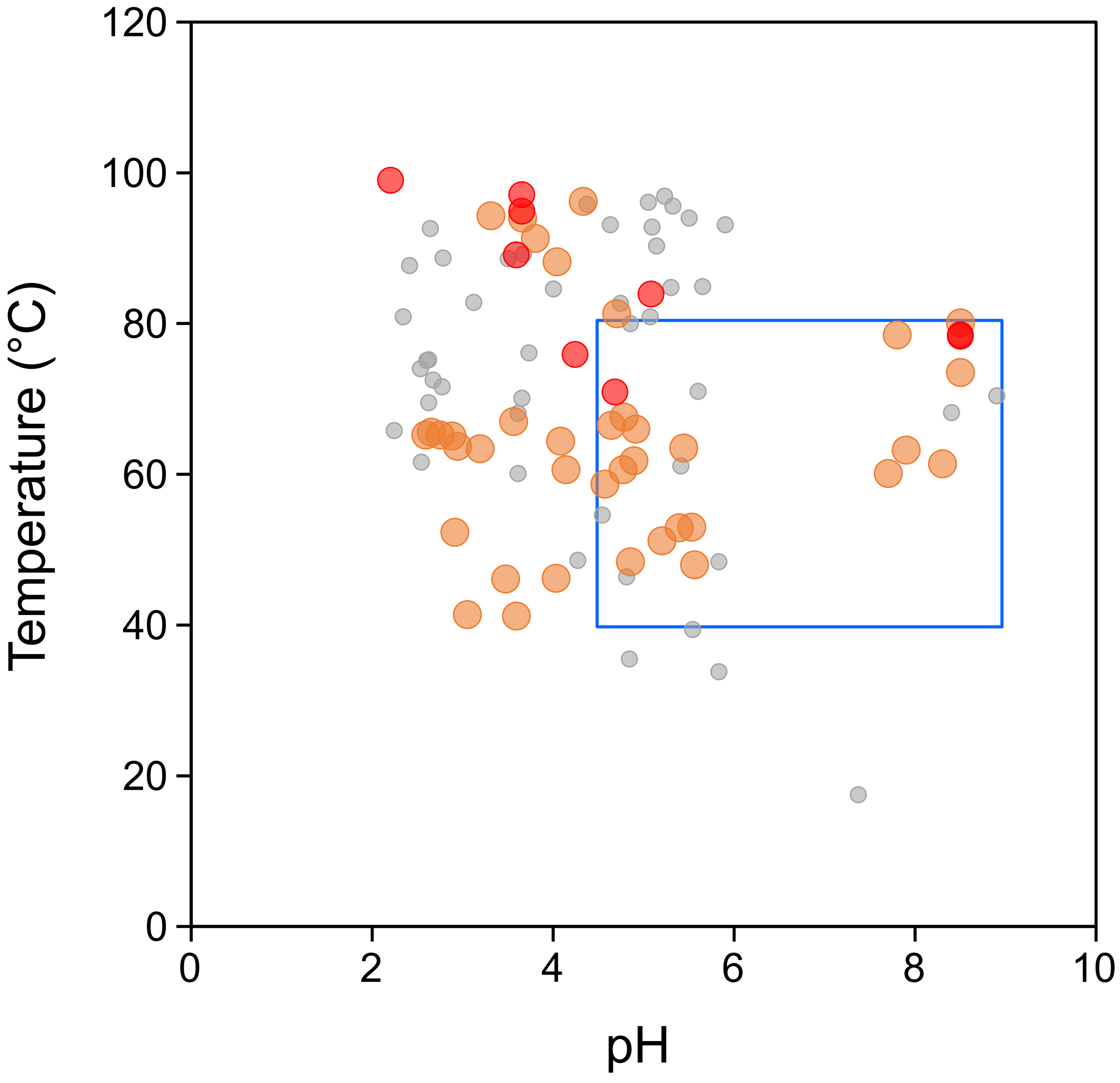


**Online Resource 8** Temperature and pH measured in hot springs. The sampling sites where any potential thermophilic hydrogenogenic carboxydotrophic phylotypes of the Phylum Firmicutes (OTU_664, OTU_1148, OTU_1160, OTU_1621, OTU_1654, OTU_1692, OTU_1749, OTU_3578, OTU_6523, OTU_6791 and OTU_8267) were detected are shown in orange circles. The sites where the relative abundance of *Caldanaerobacter subterraneus* (OTU_664), *Carboxydocella* (OTU_1654), *Carboxydothermus pertinax* (OTU_3578) or *Carboxydothermus* phylotype (OTU_1160) exceeded 0.1% are shown in red circles. The other sites are shown in gray circles. The growth range of *Caldanaerobacter*, *Carboxydocella* and *Carboxydothermus* (40-80°C, pH 4.5-9.0) are indicated by blue square.
